# Increased neutrophil-to-lymphocyte ratio is associated with unfavorable functional outcomes in acute pontine infarction

**DOI:** 10.1186/s12883-022-02969-8

**Published:** 2022-11-29

**Authors:** Mingfeng Zhai, Shugang Cao, Xinlin Wang, Yingli Liu, Feng Tu, Mingwu Xia, Zongyou Li

**Affiliations:** 1 Department of Neurology, The Affiliated Fuyang People’s Hospital of Anhui Medical University, The People’s Hospital of Fuyang, Fuyang, 236300 China; 2 Department of Neurology, The Affiliated Hefei Hospital of Anhui Medical University, The Second People’s Hospital of Hefei, Hefei, China; 3 Department of Neurology, Tongren Hospital, Shanghai Jiao Tong University School of Medicine, Shanghai, China; 4 Department of Neurology, The Affiliated Fuyang Hospital of Bengbu Medical College, Fuyang, China

**Keywords:** Pontine infarction, Neutrophil-to-lymphocyte ratio, Diffusion-weighted imaging, Functional outcomes, Inflammation

## Abstract

**Background:**

The neutrophil-to-lymphocyte ratio (NLR) is positively associated with unfavorable outcomes in patients with cerebral infarction. This study aimed to investigate the relationship between the NLR and the short-term clinical outcome of acute pontine infarction.

**Methods:**

Patients with acute pontine infarction were consecutively included. Clinical and laboratory data were collected. All patients were followed up at 3 months using modified Rankin Scale (mRS) scores. An unfavorable outcome was defined as an mRS score ≥ 3. Receiver operating characteristic (ROC) curve analysis was used to calculate the optimal cutoff values for patients with acute pontine infarction. risk factors can be predictive factors for an unfavorable outcome after acute pontine infarction.

**Results:**

Two hundred fifty-six patients with acute pontine infarction were included in this study. The NLR was significantly higher in the unfavorable outcome group than in the favorable outcome group (*P* < 0.05). Additionally, the infarct size was significantly higher in the high NLR tertile group than in the low NLR tertile group (*P* < 0.05). Multivariate logistic regression analysis revealed that the baseline National Institutes of Health Stroke Scale (NIHSS) score, NLR, platelet count, and fasting blood glucose (FBG) level were significantly associated with unfavorable outcomes 3 months after acute pontine infarction. The optimal cutoff value of the NLR for predicting the 3-month outcome of acute pontine infarction was 3.055. The negative and positive predictive values of NLR were 85.7% and 61.3%, respectively, and the sensitivity and specificity of NLR were 69.2% and 80.9%.

**Conclusions:**

We found that the NLR may be an independent predictive factor for the outcome of acute pontine infarction.

## Introduction

Ischemic stroke is associated with high morbidity, disability, and mortality rates and has become a serious threat to human health [1]. Inflammation caused by blood flow stasis, activation of intravascular leukocytes, and the release of proinflammatory mediators from ischemic endothelium, platelet granules and brain parenchyma play an important role in the occurrence and development of cerebral infarction [2, 3]. After stroke, the inflammatory response is activated, exacerbating ischemic brain damage and neurological dysfunction in secondary brain injury [4]. Recent studies have demonstrated that leukocyte counts are significantly increased in patients with acute cerebral infarction, and the increase in the number of neutrophils in these patients is positively correlated with infarct size and fatal outcome [5]. A decrease in lymphocyte count in patients with cerebral infarction is associated with poor functional prognosis [6]. It is difficult to use a single subtype index to accurately and comprehensively evaluate the prognosis of patients with cerebral infarction. The neutrophil-to-lymphocyte ratio (NLR) combines information on neutrophils and lymphocytes and is less affected than other indices by physiological factors, specimen handling and other factors; it is also inexpensive and easy to obtain and produces relatively stable results. Recently, many studies confirmed the association between this indicator and multiple diseases, including acute coronary syndrome, cancer, and neuromyelitis optica spectrum disorder, and showed that the NLR has high clinical application value [7–10]. Relevant studies have found that the NLR may be an independent predictor of unfavorable prognosis and early death in patients with acute cerebral infarction [11–13]. Therefore, an increase in the NLR may be a sensitive indicator of an increased level of inflammation after stroke.

Due to the different characteristics of the anterior and posterior circulation and differences in clinical manifestations at onset, predicting prognosis is quite challenging. The onset of anterior circulation infarction mostly presents with symptoms such as hemiparesis and aphasia, while the main manifestations of posterior circulation infarction are vertigo and dysarthria, which are relatively insidious. Pontine infarction is a common type of cerebral posterior circulation infarction; its most common clinical manifestation is motor hemiplegia, and many of its features are similar to those of basal ganglia infarction [14, 15]. Due to the relatively concentrated fiber conduction tracts affected by pontine infarction, the clinical symptoms at the time of onset are severe, and patient prognosis is unfavorable. Therefore, identifying biomarkers that can accurately predict the prognosis of patients with pontine infarction is of great significance. Previous studies have mainly focused on patients with acute cerebral infarction of the anterior and posterior circulation. Therefore, the purpose of this study was to investigate the relationship between the NLR and the clinical prognosis of patients with acute pontine infarction.

## Methods

### Subjects

This was a prospective, observational study conducted between July 2012 and December 2019, patients with acute pontine infarction who were admitted to the Hefei Affiliated Hospital of Anhui Medical University were continuously enrolled in the study. All patients were newly diagnosed with acute ischemic stroke in accordance with the clinical symptoms and neuroimaging findings listed in the World Health Organization criteria [16]. The inclusion criteria for patients were as follows: 18 years of age or older and a diagnosis of acute pontine infarction by diffusion-weighted imaging (DWI). Patients with the following conditions were excluded: (1) time between onset and admission longer than 7 days; (2) a clear history of infection or surgery before stroke; (3) liver disease or renal failure; (4) hematological disease, rheumatoid immune-related disease or tumors; (5) acute cerebral infarction other than pontine infarction; and (6) prestroke disability with a modified Rankin Scale (mRS) score > 2. The study protocol was approved by the Research Ethics Committee of the Hefei Affiliated Hospital of Anhui Medical University, and all patients or their guardians provided informed written consent to inclusion in the study.

### Clinical data

We collected demographic information, including age and sex; medical history records, including information on hypertension, diabetes, atrial fibrillation, prior stroke or transient ischemic attack (TIA), and current smoking or drinking habits; blood pressure levels on admission, including systolic blood pressure (SBP) and diastolic blood pressure (DBP); the National Institutes of Health Stroke Scale (NIHSS) score on admission; and admission to blood sampling time. Hypertension was determined based on prior use of antihypertensive drugs, SBP ≥ 140 mmHg, or DBP ≥ 90 mmHg. Diabetes was determined based on prior use of antidiabetic drugs, fasting blood glucose (FBG) ≥ 7.0 mmol/L, or 2-h postprandial blood glucose ≥ 11.1 mmol/L. Patients were considered hyperlipidemic if they had serum total cholesterol (≥ 5.2 mmol/L), triglycerides (TGs) (≥ 1.7 mmol/L), low-density lipoprotein (LDL) cholesterol (≥ 3.4 mmol/L), or a previous diagnosis of hyperlipidemia. The etiological subtypes of pontine infarction were categorized as follows: (1) vertebrobasilar large-artery disease (VLAD): patients with stenosis of at least 50% of the lumen diameter in a large artery (either a vertebral artery or the basilar artery); (2) basilar artery branch disease (BABD): patients with hypertension or diabetes, an infarct extending to the pontine surface, and no large artery occlusive disease (LAOD) or potential source of cardioembolism; (3) small artery disease (SAD): patients with hypertension or diabetes in whom the lesion (< 15 mm) did not reach the pontine surface and there was no other etiology; (4) Embolism of cardiac origin (CE): patients with potential cardiogenic sources of embolism, including nonvalvular atrial movement, left ventricular wall segmental movement disorders, intracardiac thrombus or tumor, mitral stenosis, and other minor sources; and (5) other etiologies and unknown causes [17].

### Laboratory data

Fasting blood samples were collected on the morning of the second day after admission following an overnight fast. FBG, TG, and LDL levels and other biochemical parameters were assayed using an automatic biochemical analyzer (HITACHI Automatic Analyzer 7600–020, Japan). The neutrophil and lymphocyte counts in ethylenediaminetetraacetic acid (EDTA)-anticoagulated whole-blood samples were determined within the first 24 h after admission.

### Magnetic resonance imaging (MRI) data acquisition and analysis

Within 3 days after admission, all patients underwent MRI with a 1.5 Tesla MRI scanner (Siemens Healthineers, Model: Avanto I class, Germany). The scanning protocol included DWI and an apparent diffusion coefficient sequence with 5-mm-thick sections. Three-dimensional time-of-flight (3-D TOF) magnetic resonance angiography (MRA) was acquired in the axial plane with a layer thickness of 1.4 mm. The MRI and MRA results were analyzed and mainly included the infarct lesion size at the infarct site (the maximum diameter of the infarct lesion at the largest infarct level on DWI), and a basilar artery with 50% stenosis was diagnosed as basilar artery stenosis according to the North American Symptomatic Carotid Endarterectomy Trial [18].

### Evaluation of the severity of pontine infarction and 3-month outcome

The severity of pontine infarction at admission was assessed using the NIHSS score [19]. A qualified trained neurologist followed up the short-term clinical outcomes of all enrolled patients at 3 months by telephone or appointment in the outpatient department. The follow-up ended at 3 months after acute pontine infarction or at death. The outcome was evaluated using the mRS score: an mRS score ≤ 2 was defined as a favorable outcome, and an mRS score ≥ 3 was defined as an unfavorable outcome [20].

### Statistical analyses

Data analysis was performed using SPSS version 22.0 for Windows (SPSS Inc., Chicago, IL, USA). Continuous data were assessed for normality using the Kolmogorov–Smirnov test or ANOVA. Continuous variables with a normal distribution are expressed as the mean ± standard deviation (SD). Continuous variables with a nonnormal distribution are expressed as the median (M) and interquartile range (IQR). Categorical variables are reported as absolute numbers and percentages (%). Differences in continuous variables between groups were assessed using Student’s *t* test or the Mann–Whitney U test. Differences in categorical variable distributions between groups were assessed using the *χ*^2^ test or Fisher’s exact test, as appropriate. A receiver operating characteristic (ROC) curve was used to identify the NLR cutoff that best predicted favorable and unfavorable outcomes. Variables for which the univariate analysis indicated a potential association with unfavorable outcome (*P* < 0.1) were used in the multivariate analysis. Binary logistic regression was used to verify factors independently associated with 3-month outcome. Odds ratios (ORs) and 95% confidence intervals (CIs) were subsequently calculated. Spearman’s correlation coefficient was used to determine the correlations of the NLR with NIHSS scores and mRS scores. All tests were two-tailed, and *P* < 0.05 was considered to indicate statistical significance. The figures were generated using PowerPoint and GraphPad Prism software (version 8.0).

## Results

### Clinical and demographic data

**Fig. 1:**
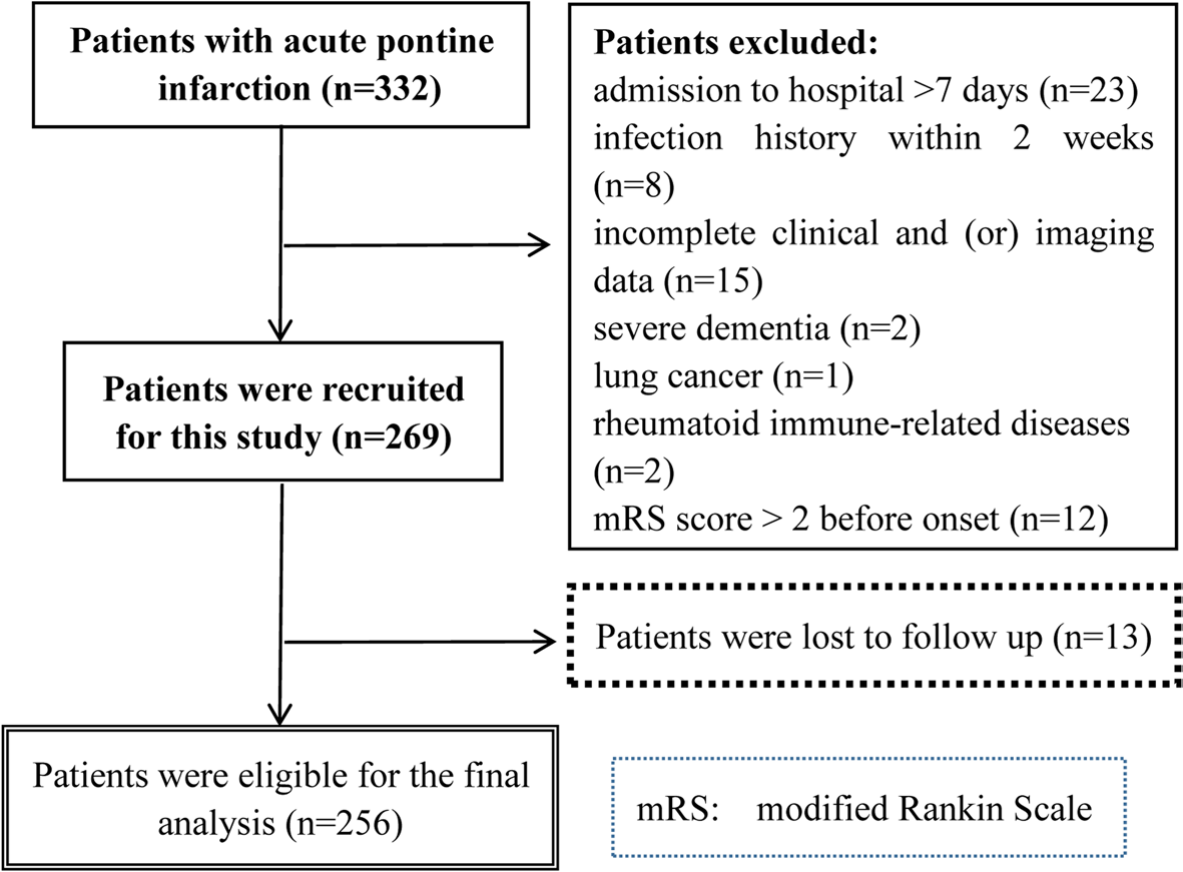
Diagram illustrating how patients with acute pontine infarction were selected for inclusion in this study

A total of 332 patients with acute pontine infarction were admitted during the study period. Of these patients, 63 were excluded based on the exclusion criteria, and 13 were lost to follow-up during the 90-day follow-up period. Ultimately, 256 eligible patients, including 155 males (60.5%), were included in this study (Fig. 1). The mean age was 66.7 ± 11.0 years, and the mean NLR was 2.4 (range 1.8–3.4).

**Fig. 2:**
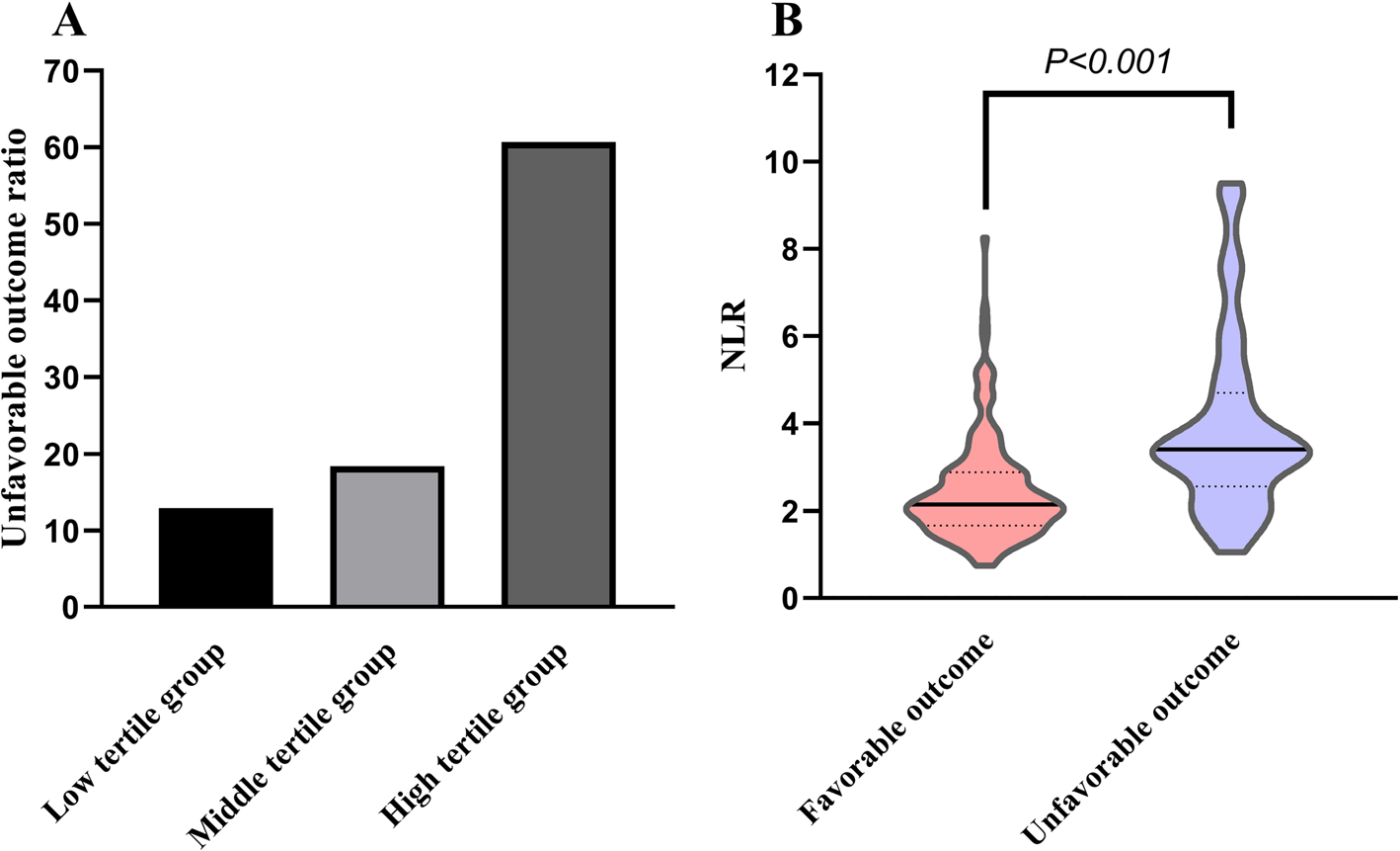
Percentage of patients with unfavorable outcomes stratified by NLR tertile (**A**). Comparison of NLR in the favorable outcome and unfavorable outcome groups (**B**)

The clinical and demographic data are shown in Table 1 and Fig. 2B. An unfavorable outcome at 3 months was found in 78 patients (30.5%). Compared with the favorable outcome group, patients with unfavorable outcomes were significantly older and had higher NIHSS scores on admission (*P* < 0.05). The neutrophil count, platelet count, NLR, platelet-to-lymphocyte ratio (PLR), LDL, high NLR tertile, and high PLR tertile values in the unfavorable outcome group were higher than those in the favorable outcome group, while the lymphocyte count was significantly lower than that in the favorable outcome group (all *P* < 0.05).

**Table 1:** Baseline clinical characteristics of patients with favorable and unfavorable 3-month outcomes

Variable	Favorable outcome (*n* = 178)	Unfavorable outcome (*n* = 78)	*P*
Age (years)	65.3 ± 11.0	69.9 ± 10.2	0.002
Male, n (%)	110 (61.8)	45 (57.7)	0.536
Hypertension, n (%)	141 (79.2)	66 (84.6)	0.312
Diabetes, n (%)	78 (43.8)	33 (42.3)	0.822
Atrial fibrillation, n (%)	4 (2.2)	4 (5.1)	0.223
Stroke history, n (%)	36 (20.2)	20 (25.6)	0.335
Smoker, n (%)	53 (29.8)	19 (24.4)	0.375
Alcohol user, n (%)	32 (18.0)	11 (14.1)	0.445
Etiological subtype, n (%)			
LAOD	39 (21.9)	20 (25.6)	0.903
CE	6 (3.4)	3 (3.8)	
BABD	72 (40.4)	33 (42.3)	
SAD	35 (19.7)	13 (16.7)	
OE	26 (14.6)	9 (11.5)	
NIHSS score	4 (3–6)	8 (7–10)	< 0.001
Admission to blood sampling time (h)	16.4 (14.3–20.8)	18.0 (14.5–21.3)	0.472
SBP (mmHg)	151.9 ± 20.1	152.6 ± 21.4	0.796
DBP (mmHg)	87.9 ± 11.6	89.5 ± 10.1	0.286
Neutrophils (10^9^/L)	4.1 ± 1.3	5.4 ± 2.0	< 0.001
Lymphocytes (10^9^/L)	1.8 ± 0.5	1.5 ± 0.5	< 0.001
Platelets (10^9^/L)	170.8 ± 45.9	186.0 ± 49.9	0.019
NLR	2.2 (1.7–2.9)	3.4 (2.6–4.7)	< 0.001
PLR	94.2 (78.9–115.3)	125.5 (96.6–143.0)	< 0.001
NLR (n,%)			< 0.001
Low tertile	74 (41.6)	11 (14.1)	
Middle tertile	71 (39.9)	16 (20.5)	
High tertile	33 (18.5)	51 (65.4)	
PLR (n,%)			< 0.001
Low tertile	72 (40.4)	13 (16.7)	
Middle tertile	64 (36.0)	22 (28.2)	
High tertile	42 (23.6)	43 (55.1)	
FBG (mmol/L)	5.6 (4.8–7.3)	5.8 (5.0–8.0)	0.078
TG (mmol/L)	1.8 (1.3–2.5)	1.8 (1.3–2.2)	0.993
LDL (mmol/L)	2.7 ± 0.8	3.0 ± 1.1	0.031
TC (mmol/L)	4.5 ± 1.0	4.6 ± 1.1	0.181
Scr (μmol/L)	67.0 (55.0–77.0)	64.5 (55.0–78.0)	0.647
BUN (mmol/L)	5.4 (4.4–6.6)	5.7 (4.6–7.4)	0.166
UA (mmol/L)	330.8 ± 88.4	313.4 ± 95.8	0.157
Infarct size (mm)	13.4 ± 4.9	14.5 ± 5.0	0.112
BA stenosis, n (%)	35 (19.7)	20 (25.6)	0.284

Values are expressed as the mean ± standard deviation; median *IQR* Interquartile range; n (%), *OR*, Odds ratio; *95% CI* 95% Confidence interval, *LAOD* Large artery occlusive disease, *CE* Embolism of cardiac origin, *BABD* Basilar artery branch disease, *SAD* Small artery disease, *OE* Other etiology, *NIHSS* National institutes of health stroke scale, *SBP* Systolic blood pressure, *DBP* Diastolic blood pressure, *NLR* Neutrophil-to-lymphocyte ratio, *PLR* Platelet-to-lymphocyte ratio, *FBG* Fasting blood glucose, *TC* Total cholesterol, *TG* Triglyceride, *LDL* Low-density lipoprotein cholesterol, *Scr* Serum creatinine, *BUN* Blood urea nitrogen, *UA* Uric acid, *BA* Basilar artery

### Comparison of clinical characteristics of acute pontine infarction according to tertiles of the NLR

**Fig. 3:**
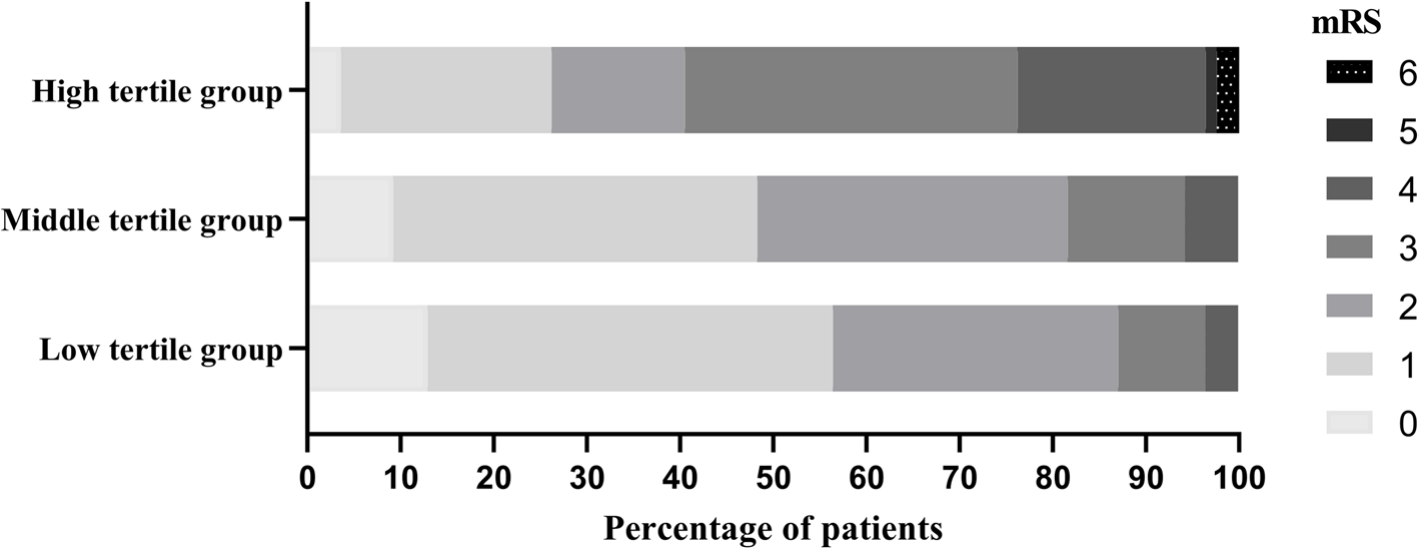
mRS score distribution of the tertile groups based on NLR

We divided the patients into tertile groups based on their NLRs on admission (low tertile group, NLR ≤ 2.03; middle tertile group, 2.03 < NLR ≤ 3.11; high tertile group, NLR > 3.11). In the low tertile group, the unfavorable outcome rate was 12.9%; in the middle tertile group, the unfavorable outcome rate was 18.4%, and in the high tertile group, the unfavorable outcome rate was 60.7% (Fig. 2A). The characteristics of these tertile groups are summarized in Table 2 and Fig. 3. Age, baseline NIHSS score, neutrophil count, and mRS score were significantly higher in the high NLR tertile group than in the middle and low tertile groups (*P* < 0.005). The lymphocyte count was lower in the high NLR tertile group than in the middle and low tertile groups (*P* < 0.005). Although significant differences in infarct size were not found among all three groups, the infarct size in the high NLR tertile group was significantly larger than that in the low NLR tertile group (*P* = 0.043).

**Table 2:** Demographic and clinical characteristics of acute pontine infarction according to NLR tertiles

	Low tertile (*n* = 85)	Middle tertile (*n* = 87)	High tertile (*n* = 84)	
Variable	NLR ≤ 2.03	2.03 < NLR ≤ 3.11	NLR > 3.11	*P*
Age (years)	65.4 ± 9.9	65.1 ± 11.5	69.6 ± 10.9	0.012
Male, n (%)	47 (55.3)	54 (62.1)	54 (64.3)	0.459
Hypertension, n (%)	63 (74.1)	75 (86.2)	69 (82.1)	0.123
Diabetes, n (%)	35 (41.2)	42 (48.3)	34 (40.5)	0.521
Atrial fibrillation, n (%)	2 (2.4)	3 (3.4)	3 (3.6)	0.881
Stroke history, n (%)	11 (12.9)	25 (28.7)	20 (23.8)	0.038
Smoker, n (%)	23 (27.1)	26 (29.9)	23 (27.4)	0.903
Alcohol user, n (%)	10 (11.8)	17 (19.5)	16 (19.0)	0.315
Etiological Subtype, n (%)				
LAOD	19 (22.4)	23 (26.4)	17 (20.2)	0.893
CE	3 (3.5)	3 (3.4)	3 (3.6)	
BABD	31 (36.5)	34 (39.1)	40 (47.6)	
SAD	18 (21.2)	15 (17.2)	15 (17.9)	
OE	14 (16.5)	12 (13.8)	9 (10.7)	
NIHSS score	5 (3–6)	5 (3–6)	7 (5–9)	< 0.001
Admission to blood sampling time (h)	16.1 (14.3–20.4)	18.3 (14.8–21.1)	17.0 (13.7–21.2)	0.278
SBP (mmHg)	151.1 ± 19.1	149.3 ± 18.2	156.0 ± 23.6	0.087
DBP (mmHg)	87.3 ± 11.2	87.4 ± 10.2	90.5 ± 11.9	0.112
Neutrophils (10^9^/L)	3.3 ± 0.9	4.2 ± 0.9	6.0 ± 1.8	< 0.001
Lymphocytes (10^9^/L)	2.1 ± 0.5	1.7 ± 0.4	1.4 ± 0.4	< 0.001
Platelets (10^9^/L)	180.2 ± 43.2	166.5 ± 47.8	179.9 ± 50.7	0.096
FBG (mmol/L)	5.6 (4.8–7.0)	5.9 (5.1–7.9)	5.6 (4.7–7.5)	0.799
TG (mmol/L)	1.9 (1.5–2.8)	1.8 (1.4–2.6)	1.6 (1.2–2.1)	0.319
LDL (mmol/L)	2.8 ± 0.7	2.8 ± 0.9	2.8 ± 1.0	0.949
TC (mmol/L)	4.7 ± 1.0	4.4 ± 1.0	4.5 ± 1.0	0.264
Scr (μmol/L)	66.0 (55.0–74.0)	66.0 (51.5–78.5)	67.5 (56.5–78.0)	0.650
BUN (mmol/L)	5.4 (4.5–6.5)	5.6 (4.3–6.5)	5.5 (4.6–7.5)	0.436
UA (mmol/L)	319.6 ± 84.6	329.8 ± 96.0	327.0 ± 92.3	0.750
Infarct size (mm)	12.8 ± 4.5	13.9 ± 5.2	13.4 ± 5.0	0.117
BA stenosis, n (%)	19 (22.4)	21 (24.1)	15 (17.9)	0.590
mRS score	1 (1–2)	2 (1–2)	3 (1–3)	< 0.001

Values are expressed as the mean ± standard deviation, median  *IQR* Interquartile range; n (%), *OR* Odds ratio, *95% CI*, 95% Confidence interval, *LAOD* Large artery occlusive disease, *CE* Embolism of cardiac origin, *BABD* Basilar artery branch disease, *SAD* Small artery disease, *OE* Other etiology, *NIHSS* National institutes of health stroke scale, *SBP* Systolic blood pressure, *DBP* Diastolic blood pressure, *NLR* Neutrophil-to-lymphocyte ratio, *PLR* Platelet-to-lymphocyte ratio, *FBG* Fasting blood glucose, *TC* Total cholesterol, *TG* Triglyceride, *LDL* Low-density lipoprotein cholesterol, *Scr* Serum creatinine, *BUN* Blood urea nitrogen, *UA* Uric acid, *BA* Basilar artery

### NLR is associated with unfavorable outcomes at 3 months after acute pontine infarction

The univariate and multivariate logistic regression models to analyze the associations between risk factors and unfavorable outcomes at 3 months. The univariate logistic regression analysis indicated that the NLR was associated with unfavorable outcomes at 90 days (OR for the high tertile group vs. the low tertile group, 10.397; 95% CI: 4.814–22.454; *P* < 0.001). After adjustment for age, number of neutrophils, number of lymphocytes, platelet count, LDL, PLR, FBG, and baseline NIHSS score, multivariate logistic regression analysis indicated that the NLR was remained associated with unfavorable outcome in patients with acute pontine infarction (OR = 5.685; 95% CI: 2.084–15.508; *P* < 0.001). In addition, baseline NIHSS score, platelet count, and FBG level were significantly associated with unfavorable outcomes at 3 months after acute pontine infarction (*P* < 0.05) (Table 3).

**Table 3:** Univariate and multivariate logistic regression analysis of outcomes

	Univariate analysis		Multivariate analysis	
	OR (95% CI)	*P*	OR (95% CI)	*P*
Age (years)	1.041 (1.014–1.069)	0.002		
Sex	0.843 (0.491–1.449)	0.536		
Hypertension	1.443 (0.707–2.947)	0.314		
Diabetes	0.940 (0.549–1.610)	0.822		
Atrial fibrillation	2.351 (0.573–9.654)	0.235		
Stroke history	1.360 (0.727–2.544)	0.336		
Smoker	0.760 (0.413–1.396)	0.376		
Alcohol user	0.749 (0.356–1.576)	0.446		
Etiological subtype				
LAOD	Ref			
CE	0.975 (0.220–4.313)	0.973		
BABD	0.894 (0.453–1.761)	0.746		
SAD	0.724 (0.315–1.668)	0.449		
OE	0.675 (0.226–1.711)	0.408		
NIHSS score	2.213 (1.795–2.728)	< 0.001	2.125 (1.698–2.658)	< 0.001
Admission to blood sampling time (h)	1.010 (0.959–1.065)	0.706		
SBP	1.001 (0.988–1.014)	0.905		
DBP	1.012 (0.989–1.036)	0.294		
Neutrophils (10^9^/L)	1.722 (1.410–2.102)	< 0.001		
Lymphocytes (10^9^/L)	0.346 (0.195–0.615)	< 0.001		
Platelets (10^9^/L)	1.007 (1.001–1.012)	0.020	1.011 (1.002–1.020)	0.015
NLR, tertile				
Low tertile	Ref		Ref	
Middle tertile	1.516 (0.659–3.490)	0.328	1.244 (0.447–3.463)	0.676
High tertile	10.397 (4.814–22.454)	< 0.001	5.685 (2.084–15.508)	0.001
PLR, tertile				
Low tertile	Ref			
Middle tertile	1.904 (0.887–4.087)	0.099		
High tertile	5.670 (2.739–11.740)	< 0.001		
FBG	1.129 (1.018–1.251)	0.021	1.217 (1.046–1.416)	0.011
TG	0.900 (0.724–1.119)	0.343		
LDL	1.397 (1.027–1.900)	0.033		
TC	1.195 (0.920–1.553)	0.182		
Scr	0.999 (0.986–1.012)	0.908		
BUN	1.111 (0.975–1.265)	0.113		
UA	0.998 (0.995–1.001)	0.158		
Infarct size	1.018 (0.964–1.075)	0.524		
BA stenosis	1.409 (0.752–2.641)	0.285		

*OR* Odds ratio, *95% CI* 95% Confidence interval, *LAOD* Large artery occlusive disease, *CE* Embolism of cardiac origin, *BABD* Basilar artery branch disease, *SAD* Small artery disease, *OE* Other etiology, *NIHSS* National institutes of health stroke scale, *SBP* Systolic blood pressure, *DBP* Diastolic blood pressure, *NLR* neutrophil-to-lymphocyte ratio, *PLR* Platelet-to-lymphocyte ratio, *FBG* Fasting blood glucose, *TC* Total cholesterol, *TG* Triglyceride, *LDL* Low-density lipoprotein cholesterol, *Scr* Serum creatinine, *BUN* Blood urea nitrogen, *UA* Uric acid, *BA* Basilar artery

### Association of NLR with baseline nihss and mrs scores in patients with acute pontine infarction

Spearman correlation analysis revealed that the NLR was positively correlated with the baseline NIHSS score (r = 0.371, *P* < 0.001) and mRS score (r = 0.368, *P* < 0.001).

### Association between NLR and 3-month outcome in patients with acute pontine infarction

**Fig. 4:**
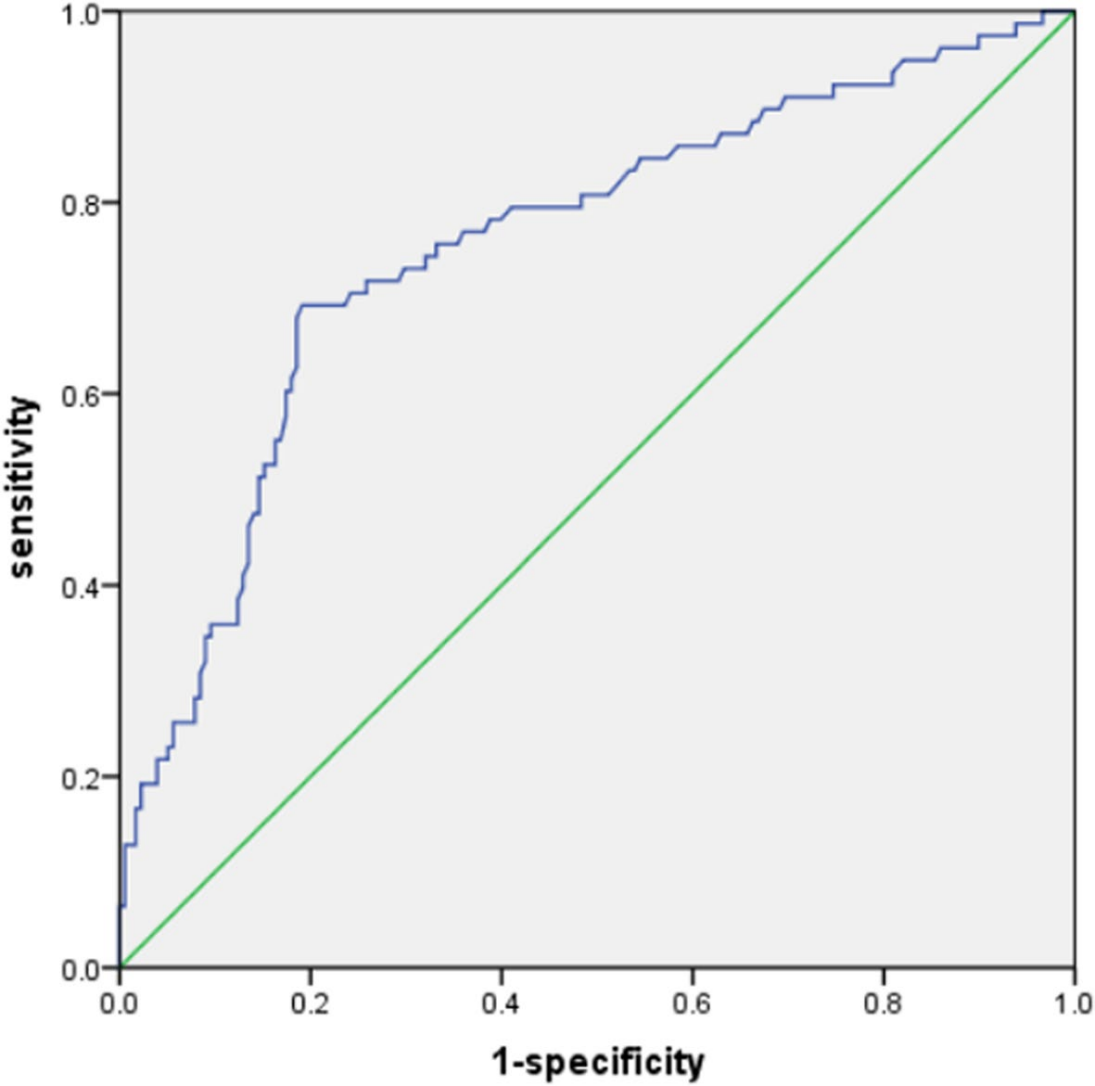
Receiver operating characteristic (ROC) curve showing the predictive value of the NLR for unfavorable outcomes (sensitivity = 0.692; 1-specificity = 0.191; NLR = 3.055; area under the curve (AUC) = 0.756)

An area under the ROC curve of 0.5 was used as the reference threshold, and the area under the NLR curve was 0.756 (95% CI: 0.689–0.823). An NLR value of 3.055 was the optimal cutoff value for the differentiation of favorable outcomes from unfavorable outcomes in patients with acute pontine infarction. The NLR exhibited negative and positive predictive values of 85.7% and 61.3%, respectively, with sensitivities and specificities of 69.2% and 80.9% for discriminating between favorable and unfavorable outcomes (Fig. 4).

## Discussion

This study followed up 256 patients with acute pontine infarction for 3 months. First, the value of the NLR in predicting the clinical prognosis of patients with acute pontine infarction was evaluated. The results indicated that a higher NLR was associated with an unfavorable short-term outcome, suggesting that the NLR may be a predictor of the 90-day prognosis of patients with acute pontine infarction. We also found significant relationships between the NLR and NIHSS score at admission and mRS score at 3 months. Therefore, an elevated NLR may be associated with unfavorable short-term prognosis in patients with acute pontine infarction.

The NIHSS has limited value in evaluating neurological deficits in patients with cerebral posterior circulation infarction. However, a common symptom of pontine infarction is pure motor hemiplegia. Therefore, the NIHSS score has greater value for evaluating the neurological deficits associated with pontine infarction than for evaluating deficits caused by posterior circulation infarctions in other areas and can be used as an important indicator to evaluate motor function deficits in patients [21]. Previous studies have indicated that baseline NIHSS scores in patients with favorable outcomes are significantly lower than those in patients with unfavorable outcomes and that a high NIHSS score is an independent risk factor for unfavorable outcomes of acute cerebral infarction [22], consistent with the results of this study.

Although a variety of pathological mechanisms are involved in poststroke injury at the cerebral ischemic site, increasing evidence indicates that inflammatory responses are closely related to injury repair and to patient prognosis and that these responses may play different roles in tissue repair and regeneration in a time-dependent manner [23, 24]. Within a few hours of the occurrence of ischemic stroke, local inflammatory responses activate neutrophils; neutrophils are the first to migrate to and increase in number at the site of vasculitis, and inflammatory mediators and cell adhesion molecules directly or indirectly aggravate the injury at the ischemic site [25]. Various subsets of lymphocytes and specific T-cell lymphocytes secrete anti-inflammatory factors, regulate the activation of glial cells, and reduce the body's immune response to cerebral ischemia [26]. In addition, lymphocytes have anti-inflammatory functions and confer endothelial protection. In the early stages of acute cerebral infarction, systemic immunosuppression can lead to a significant decrease in lymphocyte numbers; lymphocytopenia further increases susceptibility to infection [27] and adversely affects clinical outcomes. Relevant studies have shown that increased an NLR is an independent factor for unfavorable prognosis in patients with acute cerebral infarction who receive intravenous (IV) recombinant tissue plasminogen activator or endovascular treatment [28, 29]. To evaluate the relationship between the NLR and acute cerebral infarction, Chen et al. enrolled 448 consecutive patients with cerebral infarction. The study found that the NLR at hospital admission was a sensitive biomarker for predicting the prognosis of patients with acute cerebral infarction [13]. Our study also indicated that the prognosis after acute pontine infarction was worse among patients in the high NLR group and that the NLR was a predictor of an unfavorable clinical prognosis of patients with acute pontine infarction at 3 months. This finding may be related to immunosuppression and to aggravation of neurological damage caused by an increased NLR.

Previous studies have shown that neutrophils release high levels of inflammatory response mediators such as matrix metalloproteinase (MMP)-9, oxygen free radicals, cytokines, and chemokines, leading to neuronal cell death, destruction of the blood–brain barrier (BBB, and hemorrhagic transformation after acute cerebral infarction [30, 31]. Animal experiments have shown that reducing neutrophil infiltration can reduce the release of MMP-9 in the brain and that inhibition of neutrophils can reduce BBB destruction and neurological deficits after ischemic stroke [31]. Increasing the number of neutrophils through treatment with lipopolysaccharide or colony-stimulating factors can exacerbate BBB destruction and neurological deficits [32]. In addition, other factors released by neutrophils, such as reactive oxygen species, cytokines, and chemokines, can also cause tissue damage that results in BBB destruction, increased cerebral infarction volume, and nerve defects [33]. In contrast, activation of lymphocytes has a protective immunomodulatory effect and can reduce BBB destruction after stroke. The NLR reflects the pathophysiological characteristics of neutrophils and lymphocytes listed above and thereby provides comprehensive information.

This study is similar to previous studies in that infarcts were significantly higher in the high NLR tertile group than in the low NLR tertile group. This finding may be related to BBB disruption by neutrophils and to the inflammatory responses of neutrophils, resulting in increased infarct size and a greater likelihood of an unfavorable outcome. Due to the relationship between the arteries that supply the pons and the local anatomy, the corticospinal tracts within the pontine are relatively concentrated, and the nerve fibers that constitute these tracts are more likely to be involved when patients have larger infarct sizes, resulting in higher baseline NIHSS scores for these patients [34]. Accordingly, infarcts were larger in the group with unfavorable outcomes. Although the difference in infarct size between the two groups was not significant, larger infarcts led to more severe neurological deficits, resulting in worse short-term outcomes.

This study is the first to investigate the association between the NLR and the short-term outcomes of patients with acute pontine infarction. This study has some limitations. First, it is a single-center observational study. The sample size was limited, and there were regional restrictions; it lacks a large sample size and strong population representation. Therefore, some selection bias may have occurred. The results should be verified in future studies using larger, preferably multicenter, samples. Moreover, Since many patients are admitted non-emergency, the NLR sample is not determined within one hour of admission, but after 24 h. This may have an impact on the results of the study, and due to the long time span of the study, it cannot be strictly screened only at this time, but subsequent related studies can be continued based on your recommendations. Lastly, this study also did not investigate the mechanisms through which neutrophils and lymphocytes contribute to BBB damage and neurological impairment. The mechanism underlying the association between the NLR and short-term outcome after acute pontine infarction remains unclear and should be further explored in basic experiments.

## Conclusion

Our results suggest that the NLR is associated with unfavorable 3-month outcomes after acute pontine infarction and that it may represent a potential biomarker for treatment and outcome assessment. When NLR ≥ 3.055, the risk of an unfavorable outcome increases. Further large-scale research is needed before this parameter is used to predict functional outcome after acute pontine infarction.

## Data availability statement

All data generated for this study are included in the article. The datasets generated during the current study are available from the corresponding author on reasonable request.
