# Correction: Increased neutrophil-to-lymphocyte ratio is associated with unfavorable functional outcomes in acute pontine infarction

**DOI:** 10.1186/s12883-023-03089-7

**Published:** 2023-02-01

**Authors:** Mingfeng Zhai, Shugang Cao, Xinlin Wang, Yingli Liu, Feng Tu, Mingwu Xia, Zongyou Li

**Affiliations:** 1 Department of Neurology, The Affiliated Fuyang People’s Hospital of Anhui Medical University, The People’s Hospital of Fuyang, Fuyang, 236300 China; 2 Department of Neurology, The Affiliated Hefei Hospital of Anhui Medical University, The Second People’s Hospital of Hefei, Hefei, China; 3 Department of Neurology, Tongren Hospital, Shanghai Jiao Tong University School of Medicine, Shanghai, China; 4 Department of Neurology, The Affiliated Fuyang Hospital of Bengbu Medical College, Fuyang, China

**Correction: BMC Neurol 22, 445 (2022)**

**https://doi.org/10.1186/s12883-022-02969-8**

Following publication of the original article [1], the authors reported an error in the author group. Author Zongyou Li is one of the two corresponding authors of the article. The corresponding author tag was erroneously removed.

The author group has been updated above and the original article [1] has been corrected.
